# A novel 55-basepair deletion of hydroxymethylbilane synthase gene found in a Chinese patient with acute intermittent porphyria and her family: A case report

**DOI:** 10.1097/MD.0000000000012295

**Published:** 2018-09-14

**Authors:** Yi Ren, Lin-Xin Xu, Yun-Feng Liu, Chen-Yu Xiang, Fei Gao, Yan Wang, Tao Bai, Jian-Hong Yin, Yang-Lu Zhao, Jing Yang

**Affiliations:** a Shanxi Medical University; b Department of Endocrinology, The First Hospital of Shanxi Medical University, Taiyuan, China; c Epidemiology Department, University of California Los Angeles, CA, USA.

**Keywords:** acute intermittent porphyria, frameshift mutation, HMBS gene, hyponatremia, SIADH

## Abstract

**Rationale:**

Acute intermittent porphyria (AIP) is caused by hydroxymethylbilane synthase (HMBS) gene mutation.

**Patient concerns:**

A Chinese female patient with very typical AIP symptoms of severe abdominal pain, seizures, hypertension, and tachycardia, accompanied with hyponatremia, anemia, and hyperbilirubinemia.

**Diagnoses:**

She was diagnosed as AIP based on positive result of urine porphobilinogen and her clinical syndrome.

**Interventions:**

The proband was treated with intravenous glucose (at least 250 g per day) for 4 days. HMBS mutation was investigated in this family by Sanger sequencing.

**Outcomes:**

A heterozygous mutation of the HMBS gene was identified in the proband and 7 other family members. Genetic sequencing showed a deletion of 55 basepairs (C.1078_1132delGCCCATTAACTGGTTTGTGGGGCACAGATGCCTGGGTTGCTGCTGTCCAGTGCCT) including the stop codon position, leading to frameshift mutation. The mutation has not been documented in current gene databases. Further prediction of mutated protein structure suggests that the mutation is likely to produce prolonged peptide with structural change and less stability.

**Lessons:**

Physicians should pay attention to AIP attack in patients with suspected symptoms and make use of genetic testing to increase identification of mutated HMBS gene carriers for further preventive strategy.

## 1 Introduction

Acute intermittent porphyria (AIP, MIM # 176000) is an autosomal dominant inheritance disease caused by the gene mutation of hydroxymethylbilane synthase (HMBS, also known as porphobilinogen deaminase). HMBS is the third enzyme in the heme synthesis pathway that catalyzes 4 porphobilinogen (PBG) into the hydroxymethylbilane.^[1]^ Mutation of the HMBS gene leads to HMBS deficiency and accumulation of porphyrin precursors including δ-amino levulinic acid (ALA) and PBG. In the presence of some medication and other provoking factors like stress and fasting, these redundant precursors will trigger the acute and severe onset of AIP. It is crucial for physicians to make a timely diagnosis to prevent life-threatening progression of AIP. However, diagnosis of AIP is still a huge challenge in many countries,^[2–6]^ mainly due to variable clinical manifestations and/or low penetrance. Clinical diagnosis of AIP is mostly based on raised levels of PBG in urine and serum.

Currently genetic sequencing is being wildly used to detect the HMBS gene mutations. More than 400 mutations of the HMBS gene have been identified in the world. We present here a novel heterozygous deletion mutation of the HMBS gene found in a Chinese female patient with AIP and her family. A removal of 55 basepairs (bps, c.1078_1132del) results in frame shift and 3′UTR (untranslated regions) destruction.

## 2 Methods

### 2.1 Subjects

One AIP patient and 9 family members from 3 generations were included in our study. The study was subject to approval by the ethics committee of First Hospital of Shanxi Medical University and informed consent was obtained from all participants.

### 2.2 Case presentation

**Figure 1:**
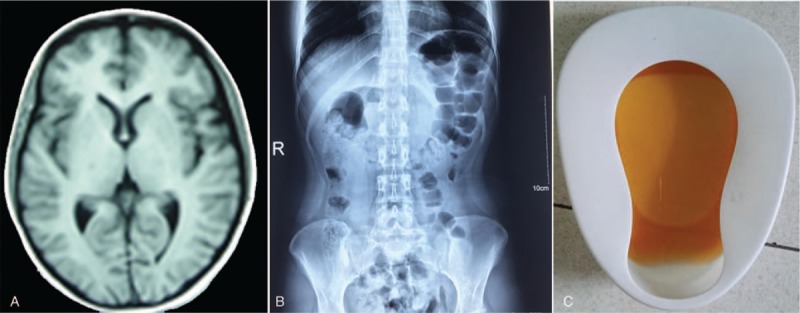
Clinical materials of the proband. (A) Brain magnetic resonance imaging (MRI): normal, (B) Abdomen X-ray: intestinal tympanites, (C) brown urine.

The proband was a 21-year-old Chinese female (Han ethnicity). On April 1, 2016, she suffered from severe abdominal pain with nausea and vomiting after having some ice cream for lunch. She was given supportive treatment against “gastroenteritis” and soapy-water enema therapy against “incomplete intestinal obstruction” by local hospitals. On April 7th, she had an epileptiform seizure accompanied by hyponatremia (Na: 103 mmol/L, normal: 130–150 mmol/L), hypokalemia (K: 2.85 mmol/L, normal:3.5–5.5 mmol/L), and hyperbilirubinemia (total bilirubin: 68.19 μmol/L, normal: 2–20 μmol/L; indirect bilirubin: 58.66 μmol/L, normal: 0–14 μmol/L). In the ensuing days, she manifested consciousness disorder (drowsiness, illusion, and confusion), persistent lower limb weakness and pain, sinus tachycardia, and hypertension in addition to recurrent abdominal pain. The imaging examination of her brain and abdomen showed normal results, except for intestinal tympanites and cholecystolithiasis (Fig. 1A, B). However, monitoring of her blood testings indicated further deterioration, including anemia (hemoglobin: 76 g/L, normal: 115–150 g/L), hypohepatia (alanine aminotransferase: 111 U/L, normal: 0–40 U/L), and elevated pancreatic enzyme (lipase: 682 U/L, normal: 0–190 U/L). On April 12th, the patient was transferred to our hospital. In the following 20 days, her condition was gradually improved after water intake restriction. In the meanwhile, she manifested brown urine (Fig. 1C), indicating diagnosis of AIP, which was finally confirmed by the elevated level of urine PBG and the negative result for urine lead. On December 30th, she presented in our hospital again with drastic abdominal pain and mild consciousness disorder. After 4 days’ treatment with intravenous glucose (at least 250 g per day), her symptoms were alleviated.

The patient was born from a nonconsanguineous family. She has a history of constipation from childhood and shortened menstrual cycles. Clinical characters of her family members are showed in Table 1. Of 9 family members, 6 had refractory constipation, 3 had recurrent abdominal pain. Almost all the female members had irregular menstruation.

**Table 1:**
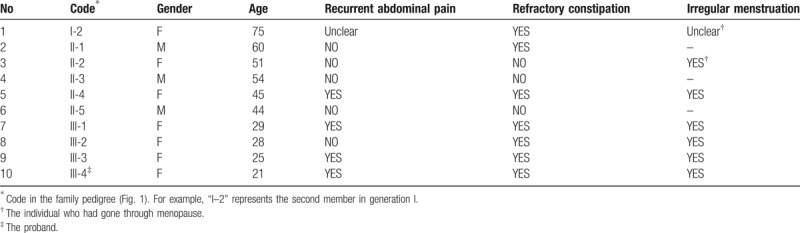
Clinical characters of 10 family members.

### 2.3 Gene sequencing and bioinfomatics analysis

HMBS gene is located at chromosomal region 11q23.3, containing 15 exons and producing 2 transcripts initiated from 2 different promotors. The upstream promotor initiates the housekeeping transcript (NM_000190.3) including exons 1 and 3 to 15, which encodes a protein of 361 amino acid (aa) in all tissues, The downstream promotor is responsible for initiating the erythroid transcript containing exons 2 to 15, which encodes a protein of 344 aa, and is not active in nonerythroid cells.

Genomic DNA was extracted from peripheral blood specimens and sent to Macro-micro-test Company. Mutational analysis for all exons and intron-exon junctions of the HMBS gene (NM_000190.3) was carried out by directly sequencing method on ABI 3730 Genetic Analyzer. We investigated whether the newly found mutation had been reported previously by searching for online databases in Human Gene Mutation database (HGMD, http://www.hgmd.org), Exome Aggregation Consortium (ExAC, http://exac.broadinstitute.org), and 1000 Genomes Project (1000G, http://www.1000genomes.org ). For the newly found variation, we made mutation analysis including mutation type, aas conservation analysis, and possible mutant protein structure, using ORFfinder Software (https://www.ncbi.nlm.nih.gov/orffinder) and PredictProtein (PP) software (https://www.predictprotein.org). Furthermore, we made pathogenicity evaluations according to Genetic Variant Interpretation Tool (http://medschool.umaryland.edu/Genetic_Variant_Interpretation_Tool1.html) based on the ACMG/AMP Standards and Guidelines.^[7,8]^

## 3 Results

**Figure 2:**
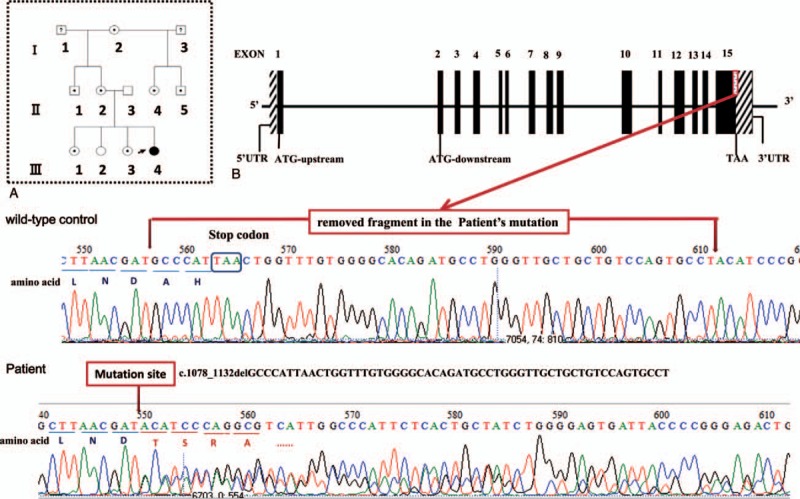
Gene sequencing analysis. (A) Family pedigree. Three generations (I, II, and III) were recruited in our study. A dark arrow indicates the proband. Square symbols indicate male members. Circle symbols indicate female members. Symbols with a question mark represent family members who are not recruited in our study. Symbols with black color represent family members who carry the mutation and experienced clinically confirmed disease. Symbols with a dot in the middle represent family members who carry the mutation but without clinical disease. (B) The novel mutation in HMBS gene. The mutation shows that 55 bps including original termination codon are removed. The deletion of 55 bps results in frame shift and 3′UTR destruction of HMBS gene. bp = basepair, HMBS = hydroxymethylbilane synthase, UTR = untranslated regions.

**Figure 3:**
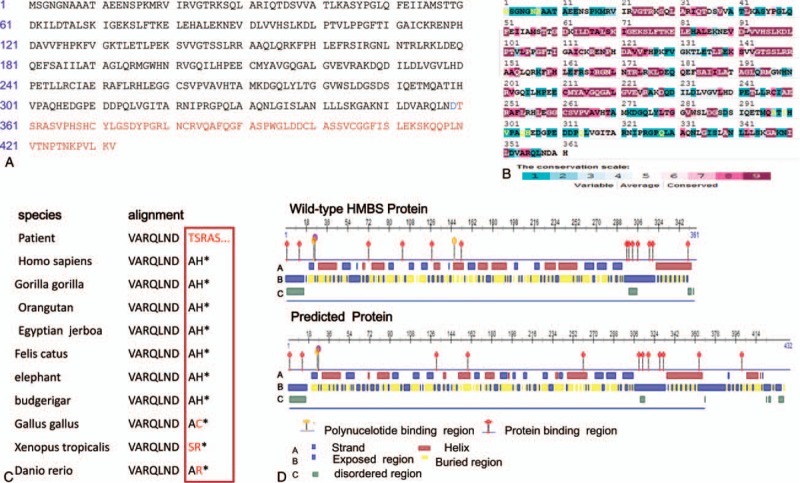
Mutation pathogenicity analysis. (A) ORFfinder software predicts that the novel mutation gene translates a prolonged peptide (432 aas). The predicted protein contains 359 of 361 aas of HMBS, followed by 73 extra aa (shown in red) that are not identical to the any part of wild-type HMBS protein. (B) HMBS protein (P08397) conservation analysis. Result from PredictProtein software. The conservation scale is indicated by different colors labeled with number 1 to 9 (1 – lowly conserved sequence, 9 – highly conserved sequence). The result reveals that both 360th and 361th aa conservation scores are 5 (moderately conserved). (C) Evolutionary conservation of aa residues across different species. (D) PredictProtein software reveals that there is obvious difference between wild-type and predicted protein in strand, helix, polynucelotide binding region, protein binding region, etc. aa = amino acid, HMBS = hydroxymethylbilane synthase, UTR = untranslated regions.

The gene sequencing result shows that a heterozygous variant of the HMBS gene in exon 15 (C.1078_1132delGCCCATTAACTGGTTTGTGGGGCACAGATGCCTGGGTTGCTGCTGTCCAGTGCCT) was found in 8 participants (labeled with black dot and the proband (Fig. 2A). A deletion of 55 bps leads to a frameshift mutation with original stop codon loss and 3′UTR destruction (Fig. 2B). ORFfinder software predicts that new stop code presents in the end of Exon 15. The mutation is not found in HGMD, EXAC, or 1000G, and was thought to be a novel deletion mutation in HMBS gene. The mutated gene is liable to translate an extended protein (Fig. 3A). We analyzed protein sequence conservation scale of the wild-type protein and secondary structure of the predicted protein. The 2 residues close to original stop codon are semi-conserved in HMBS protein (Fig. 3B, C). The predicted protein with prolonged peptide leads to an obvious change in strand and helix. Some previous binding sites of protein and polynucelotide are also altered (Fig. 3D). The change in structure probably affects predicted protein's activity and stability. What is more, the interpretation of the mutation we found is “pathogenic,” according to Genetic Variant Interpretation Tool. These findings force use to speculate that the novel mutation should be responsible for AIP attack. We have submitted these data about the novel mutation to ClinVar (https://www.ncbi.nlm.nih.gov/clinvar/submitters/506477).

## 4 Discussion

Individuals with AIP are easily misdiagnosed because of nonspecific and variable manifestation. Patients in acute attacks typically present 3 dominated clinical manifestations (acute abdominal pain, peripheralor spinal neuropathy, and psychiatric disorders), resulted from the redundancy of the porphyrin precursors. A total of 95% patients with acute onset have severe but poorly localized abdominal pain and may be wrongly diagnosed as “acute abdomen,” such as appendicitis, cholecystitis, etc.,^[5,9]^ as is in our case. The patient's first chief complaint was acute abdominal pain with other typical symptoms including nausea, vomiting, constipation or diarrhea, tachycardia, and hypertension. She progressed hyponatremia as a result of syndrome of inappropriate antidiuretic hormone, which is also common in AIP acute attack. It is very important to make timely diagnosis and timely treatment. In our case, the patients were diagnosed based on urine PBG tests and was quickly relieved after the right diagnosis and treatment.

AIP patients can be classified as overt or latent according to clinical presentation. Clinically manifest (or overt) AIP is defined as the individuals who are symptomatic at present or who are in remission following an acute onset. Latent (or presymptomatic) AIP is defined as the individuals who are at high risk of developing acute attacks. The majority of latent AIP will remain asymptomatic or mildly uncomfortable throughout their lives. However, life-threatening attacks can be triggered or deteriorated by morbid conditions such as malnutrition and infection, or some normal events such as starvation, alcohol intake, pregnancy, or certain medication, such as phenobarbitone.^[10]^ In our case, the 7 relatives of the patients with mutated gene only have no or very mild symptoms and the patient's acute attack is triggered by eating cold food. Current prophylactic efforts are focused on identifying AIP individuals in order to help them avoid the environmental triggering factors.

Genetically, AIP is an autosomal dominant disease caused by HMBS gene mutation. Most of the symptomatic patients were reported in Europe, especially in Scandinavian countries.^[11]^ An estimated prevalence of symptomatic AIP in European countries excluding Sweden is about 5.4/1,000,000.^[11]^ Some studies based on genetic testings reveal that its prevalence is substantially underestimated because high-risk carriers of AIP present extremely low penetrance in Caucasian population.^[12,13]^ According to HGMD, more than 400 distinct mutations in HMBS have been reported in the world. Most of the HMBS mutations have a low frequency in independent AIP families.^[14]^ In other words, the majority of them are not shared by the other families. That is so called “family specific.” The epidemic is still unclear in Chinese population due to low diagnosis rate. An early study revealed a high misdiagnosis rate in Mainland China.^[15]^ A small study from Taiwan identified 25 HMBS mutations, 11 of which were newly found.^[16]^ Some recent reports provided basic clue to understand the diagnosis and treatment situation for Chinese AIP patients. Very excitingly, gene analysis assists Chinese physicians identify AIP patients and potential carriers. C.806 C>G (p. T269R), IVS11–2A→G, p.R173Q, c.973C>T (p.R325X), and c.1071delT mutations were identified in the Chinese population.^[6,17–19]^ IVS2–2AgG, c.655G>C (p.A219P), and c.988G>C (p.Ala330Pro) mutations were newly found.^[18,20,21]^ At the same time, these scattered reports reveal the fact that the biochemistry or gene testing on AIP cannot be universal yet in China, in accordance with the phenomena that most Chinese doctors may not be familiar with varied clinical manifestations of AIP.

We found a heterozygous mutation of the HMBS gene. The novel mutation is located at the last exon (Exon 15) – a crucial exon covering coding sequence, termination codon, and 3′UTR. A nonsynonymous variant c.1075G>A (p.D359N) that is very close to the mutation site we found was previously recorded as a disease-causing gene.^[12,22]^ Similar to our findings, a frameshift mutation C.1073delA with original stop codon loss presumably creates an elongated protein with enzyme activity defection.^[23]^ We presume that the mutation marked with original termination codon loss and frameshift can produce malfunctional protein due to longer peptide tail and subsequently alter secondary protein structure. We found difference between wild type protein and the mutated protein, not only in the primary structure but also in the conformations (Fig. 3): moderately conserved A360 and H361 are removed; additional 73 aas are followed by; remarkable change in C-terminal helix; and shift of polynucelotide binding region, and protein binding region. Although the mutated protein contains all the structural domains that have been known to be necessary for the enzymatic function of HMBS, an additional tail probably affects its activity and stability. In further investigation, we plan to test enzymatic activity of HMBS of the patient, and the level of mRNA and protein caused by the novel mutation.

In conclusion, the AIP family in our study was found to have a novel 55-bp deletion mutation of the HMBS gene, which was very likely to cause HMBS deficiency by altering protein structure. In China, medical professionals should pay attention to AIP attack in patients with suspected symptoms and make use of genetic testing to increase identification of mutated HMBS gene carriers for further preventive strategy.

## Acknowledgments

The authors thank the Natural Science Foundation of China [No. 81270882] and the Natural Science Foundation of Shanxi Province, China [No. 2014011043-3] for the support. The authors also thank Jun Jiang working in Macro-micro-test Company for her help.

## Author contributions

**Conceptualization:** Yi Ren, Fei Gao, Jing Yang.

**Data curation:** Chen-Yu Xiang, Tao Bai, Jian-Hong Yin.

**Funding acquisition:** Jing Yang.

**Investigation:** Yan Wang.

**Software:** Yi Ren.

**Validation:** Jing Yang.

**Writing – original draft:** Yi Ren.

**Writing – review & editing:** Lin-Xin Xu, Yun-Feng Liu, Yang-Lu Zhao.

Yi Ren orcid: 0000-0002-1163-3642
